# Chronic imaging through “transparent skull” in mice

**DOI:** 10.1371/journal.pone.0181788

**Published:** 2017-08-16

**Authors:** Anna Steinzeig, Dmitry Molotkov, Eero Castrén

**Affiliations:** 1 Neuroscience center, University of Helsinki, Helsinki, Finland; 2 Biomedicum Imaging Unit, University of Helsinki, Helsinki, Finland; Radboud University Medical Centre, NETHERLANDS

## Abstract

Growing interest in long-term visualization of cortical structure and function requires methods that allow observation of an intact cortex in longitudinal imaging studies. Here we describe a detailed protocol for the “transparent skull” (TS) preparation based on skull clearing with cyanoacrylate, which is applicable for long-term imaging through the intact skull in mice. We characterized the properties of the TS in imaging of intrinsic optical signals and compared them with the more conventional cranial window preparation. Our results show that TS is less invasive, maintains stabile transparency for at least two months, and compares favorably to data obtained from the conventional cranial window. We applied this method to experiments showing that a four-week treatment with the antidepressant fluoxetine combined with one week of monocular deprivation induced a shift in ocular dominance in the mouse visual cortex, confirming that fluoxetine treatment restores critical-period-like plasticity. Our results demonstrate that the TS preparation could become a useful method for long-term visualization of the living mouse brain.

## Introduction

Currently available imaging methods create unique opportunities for studying cortical structure and plasticity. Providing improved spatial resolution and the possibility to observe large areas of the cortex, optical tools have become a valuable alternative to standard methods in plasticity experiments, such as single-unit recordings and visual-evoked potentials [1,2,3].

Diverse approaches have been developed to provide optical access to the cerebral cortex. Implantation of a cranial window (CW) is a broadly utilized method for chronic imaging of cortical responses [4]. Despite the optimal optical resolution that the CW offers, this method nevertheless has limitations. There are two ways to implant a CW; one includes drilling a hole in the skull and the other requires only bone thinning. The craniotomy required for window embedding may lead to dura regrowth, increased dendritic-spine turnover, and an inflammatory response [5,6]. Accordingly, craniotomy usually necessitates a 3–4 week delay between surgery and imaging [7]. On the other hand, bone thinning provides a relatively small area for imaging, may require re-thinning, and is mechanically vulnerable [7]. Furthermore, the area covered by a CW is limited, and it is difficult to insert a window into certain parts of the skull, such as the somatosensory area representing a front limb. Moreover, the rigidity of a CW is an obstacle for manipulations such as viral injection or drug administration.

Due to the limitations of the CW approach, a growing number of alternative methods for chronic and less invasive optical imaging have recently emerged. These methods include less penetrative skull thinning techniques [7,8], including a more advanced thinned skull reinforced by a glass window preventing bone regrowth [9,10], a silicon-based CW that allows a larger area for observation [11], and a non-invasive clear skull-cap method [12].

The visual system is a well-established model for plasticity studies. Occlusion of one eye for a short period during early postnatal life enhances the response of the primary visual cortex to the open eye at the expense of the occluded eye, a process called ocular dominance (OD) plasticity [13,14]. This ability of the nervous system to change in response to experience was long thought to be restricted to a brief postnatal period of time, known as the critical period [1,15,16]. However, recent studies have revealed that a critical-period-like OD plasticity can be reinstated in adult animals by a number of treatments, such as chronic antidepressant treatment or environmental enrichment [17,18,19,20]. Thus, the process of critical period plasticity can be investigated by reopening a natural period of OD plasticity in adulthood, even if it had already closed earlier in development.

Here, we provide a detailed description of the “transparent skull” (TS) technique, an inexpensive, rapid, and comparatively non-invasive method for preparation of the skull for chronic imaging in mice. Furthermore, we demonstrated the utility of this method for an intrinsic optical imaging experiment, and compared the data obtained against CW data. In addition, we applied the TS technique for the measurement of fluoxetine-induced plasticity in the mouse visual cortex. Our data indicates that the TS preparation provides sufficient quality for optical imaging and can serve as an alternative to CW implantation.

## Materials and methods

### Animals

Female C57BL/6J Rcc Hsd mice used in all experiments were obtained from Envigo (Harlan Labs, UK). Animals were raised in standard conditions (temperature 22°C, 12-hour light/dark cycle) with fresh food and water available *ad libitum*. The age of the animals for experiments with fluoxetine-induced plasticity was at least 18 weeks at the beginning of monocular deprivation, as natural critical-period plasticity has ended at this time [16]. All experiments were performed according to institutional guidelines and were approved by the County Administrative Board of Southern Finland (License number: ESAVI/7551/04.10.07/2013). All efforts were made to minimize animal suffering.

### Surgical procedures

For surgical anesthesia, animals were injected with a mixture of Fentanyl (Hameln, Germany) 0.05 mg/kg, Midazolam (Hameln, Germany) 5 mg/kg, and Medetomidine (Orion Pharma, Finland) 0.5 mg/kg. Additionally, Carprofen 5 mg/kg (ScanVet, Nord Ireland) was administrated s.c. for postoperative analgesia.

While under anesthesia, animals were kept on a heating mat at a temperature of 37°C to maintain normal body temperature and fixed into a stereotaxic frame. The eyes were protected from drying with eye gel (Viscotears, Alcon, UK).

The scalp was first shaved, disinfected with 70% ethanol and iodine solution, and then removed with spring scissors. The periosteum was removed by gentle scratching with an eye scalpel, and lateral muscles were retracted to expose sufficient skull surface. Debris was blown away during scratching with an airstream from a custom-made air pump. The skull surface was prepared so as to remain completely dry and clean. After cleaning, acetone was applied for surface degreasing. The airstream also served to prevent acetone penetration into the bone.

For the TS method, a thin layer of cyanoacrylate glue Loctite 401 (Henkel, Germany) was applied with a metal stick and allowed to dry for approximately 15 minutes. Colorless acryl powder (EUBECOS, Germany) was mixed with methyl methacrylate liquid (Densply, Germany) to a nail-polish-like consistency, and a thin layer was applied to the skull with a painting brush. The first layer was allowed to dry for about 40 minutes before the second layer was applied. The layers were left to dry overnight.

The next day, the acrylic layer was polished only in the region of interest (ROI) with fine acryl polishers (Shofu inc., Germany). A metal bar holder was first glued to the skull surface and then covered with a mixture of cyanoacrylate glue and dental cement (Densply, Germany). Transparent nail polish was added to the inside the metal holder above the ROI. To test the possibility of injections through the TS, a small hole was drilled above the ROI. The CW was implanted in accordance with a previously published protocol [4].

### Monocular deprivation

The left eye of the animals used for the plasticity experiment was sutured shut with 3 mattress sutures under anesthesia as described above. During seven days of deprivation, all animals were checked daily, and those demonstrating spontaneous reopening were excluded from the experiment.

### Injection through the TS

To drill the hole in the skull, we used a dental drill (HP4-917, Foredom Electrics, USA) with a custom-made drill bit approximately 0.2 mm in diameter. The injection spot was targeted with intrinsic signal optical imaging. We injected 0.5 μl saline in the binocular area of the primary visual cortex with custom-made beveled borosilicate glass needles with a 20–30 μm tip diameter attached to a 10 μl syringe (WPI Nanofil) and microsyringe pump (WPI). With a stereotaxic manipulator, we positioned the injection needle 0.9 mm below the skull surface and performed an injection.

### Fluoxetine treatment

For the plasticity restoration experiments, the animals were treated for four weeks with 80 mg/l of Fluoxetine (fluoxetine-hydrochloride) dissolved in their drinking water. The control group received regular tap water. Water consumption was monitored twice a week in both groups. No decline in drinking volumes were observed in the fluoxetine group (S1 Fig).

### Optical imaging

We recorded intrinsic signal responses from the primary visual cortex of the right hemisphere according to a previously described protocol [21] that has been modified for measurement of OD plasticity [3]. Intrinsic signals were recorded with a Dalsa 1M30 CCD camera (Teledyne-Dalsa, Canada) with a tandem macro objective (50 x 135 mm, Nikon, Japan). We used continuous-periodic stimulation with continuous synchronized data acquisition for the processing of the intrinsic signals. The visual stimulus was represented by a drifting thin horizontal bar (2° wide) displayed on a high refresh rate stimulus monitor (background luminance of 5 cd/m^2^; stimulus luminance 16 cd/m^2^). The bar moved upwards with a temporal frequency of 1 cycle/8.3 s (0.125 Hz) and a spatial frequency of 1/80 deg. To restrict the stimulus presentation to the binocular area of the primary visual cortex, the bar was shown only in the central part of the screen (-15° to +5°, since we always imaged the right visual cortex). For the imaging experiment, the animals were anesthetized with 1.2% isoflurane in a 1:2 mixture of O_2_:air, placed on a heating pad facing the stimulus monitor located within 25 cm of front of the mouse, with a vertical midline aligned to the animal’s snout. The head of the animal was tightly fixed during the optical recordings, and the eyes remained in a stable position throughout the experiment.

To map a vascular pattern, the brain surface was first illuminated with green light (540±20 nm). After acquisition of the surface image, the camera was focused at 600 μm below the cortical surface and then illuminated with red light (625±10 nm) to record the intrinsic signals. An additional red filter (longpass 590 nm) was placed between the brain surface and the camera to prevent light contamination from the screen during the intrinsic signal acquisition. The frames were collected independently during left and right eye stimulation (the other eye was kept closed with a patch) for 300 sec at a rate of 30 fps and stored as a 512x512 pixel image after spatial binning of the camera images.

### Data analysis

Cortical maps were computed based on the acquired frames using Fourier decomposition to extract the signal from biological noise using an analysis software package for continuous recording of optical intrinsic signals (VK Imaging, USA) [21]. Briefly, the frames were averaged temporally to remove slow hemodynamic artifacts. The magnitude of the intrinsic response (presented as fractional changes in reflectance x 10^4^) was utilized in this method to calculate the primary visual cortex (V1) activation via ipsilateral and contralateral stimulation and an ocular dominance index (ODI). The ODI was calculated as previously described [3]. For this calculation, the ipsilateral magnitude map was smoothened with a low-pass filter with uniform kernel of 5x5 pixels. The smoothened map was then thresholded (30% of maximum response) and used as a mask to select the binocularly responding region. The ODI was then calculated for every pixel within the binocularly responding region based on the formula (C-I)/(C+I), where “C” refers to the response magnitude of the contralateral eye and “I” to that of the ipsilateral eye. For each animal, several ODIs were quantified and then averaged. Positive ODI values represent contralateral dominance, negative represent ipsilateral dominance, while ODI values of 0 correspond to equally strong contralateral and ipsilateral eyes.

### Statistical analysis

Groups were compared using one-way and two-way ANOVA and Student’s t-test. For post-hoc analysis, we performed Sidak’s and Tukey’s test for multiple comparison. Significance levels were set as *P<0.05; **P<0.01; ***P<0.001. Data are presented as means ± SEM. Detailed statistics are described in supplementary table (S1 Table).

### Detailed protocol for transparent skull surgery

#### Surgical equipment

- Stereotaxic frame for small animals (World Precision Instruments, 502063, USA)
- Heating pad with temperature controller (Supertech Instruments, TMP-5b, UK)
- Heating mat (M.A.T. Solutions B.V., Netherlands)
- Surgical light source (Highlight 2001, Olympus, Germany)
- Air-blow system (custom-made)
- Stereoscope (Nikon, SMZ745T)
- Hand drill (HP4-917, Foredom Electrics, USA)
- Drill bit polishers (AcryPoint PC2, Shofu)
- Syringes and needles

#### Drugs

- Fentanyl (Hameln, Germany, 50 μg/ml)
- Midazolam (Hameln, Germany, 5 mg/ml)
- Medetomidine (Orion, Finland 1 mg/ml)
- Saline, sodium chloride 9 mg/ml (B Braun, Germany)
- Carprofen (ScanVet, Nord Ireland)
- Eye-protective gel (Viscotears)
- Betadine (Leiras, Finland)
- Lidocaine c. adrenalin 20mg/ml (Orion Pharma, Finland)

#### Surgical instruments

- Spring scissors (15019–10; 91500–09 Fine Science Tools, Canada)
- Curved forceps (11052–10, Fine Science Tools, Canada)
- Eye scalpel (10035–12, Fine Science Tools, Canada)
- Metal stick (CA 08005, Medicon, Germany)
- Silk thread (K-802, Ethicon, USA)

#### Other materials

- Acryl powder (EUBECOS, Germany)
- Dental cement (Densply, Germany)
- Methacrylate liquid (Densply, Germany)
- Transparent nail polish (#72180, Electron Microscopy Sciences)
- Cyanoacrylate glue (Loctite 401, Henkel, Germany)
- Super glue gel (Loctite Power Flex, Henkel, Germany)
- Metal head holders (Neurotar, Finland)
- Cotton swabs
- Brush
- Acetone
- Ethanol

#### Critical points

- **Window quality.** Avoid any debris, fur, or roughness in the ROI that can affect adhesion of the covering layers and quality of the imaging.
- **Metal holder stability.** Expose enough of the skull area to provide sufficient space for adhesion. Use an adequate amount of dental cement where possible; the holders could otherwise become detached.
- **Eye protection.** Always protect the animals’ eyes during surgery. The eyes dry out very quickly, which results in blindness. Use eye gel generously; reapply when needed. Keep dental cement from spreading into the eyes.

#### Steps: day 1

1 Prepare anesthesia: mix Fentanyl 0.05 mg/kg, Midazolam 5 mg/kg, and Medetomidine 0.5 mg/kg diluted in saline. For 10 ml of solution, dilute 1 ml Fentanyl, 1 ml Midazolam, and 0.5 ml Medetomedine in 7.5 ml of saline.

**TIPS**: Always check drug concentration on package; concentration may vary depending on manufacturer.

**Fig 1:**
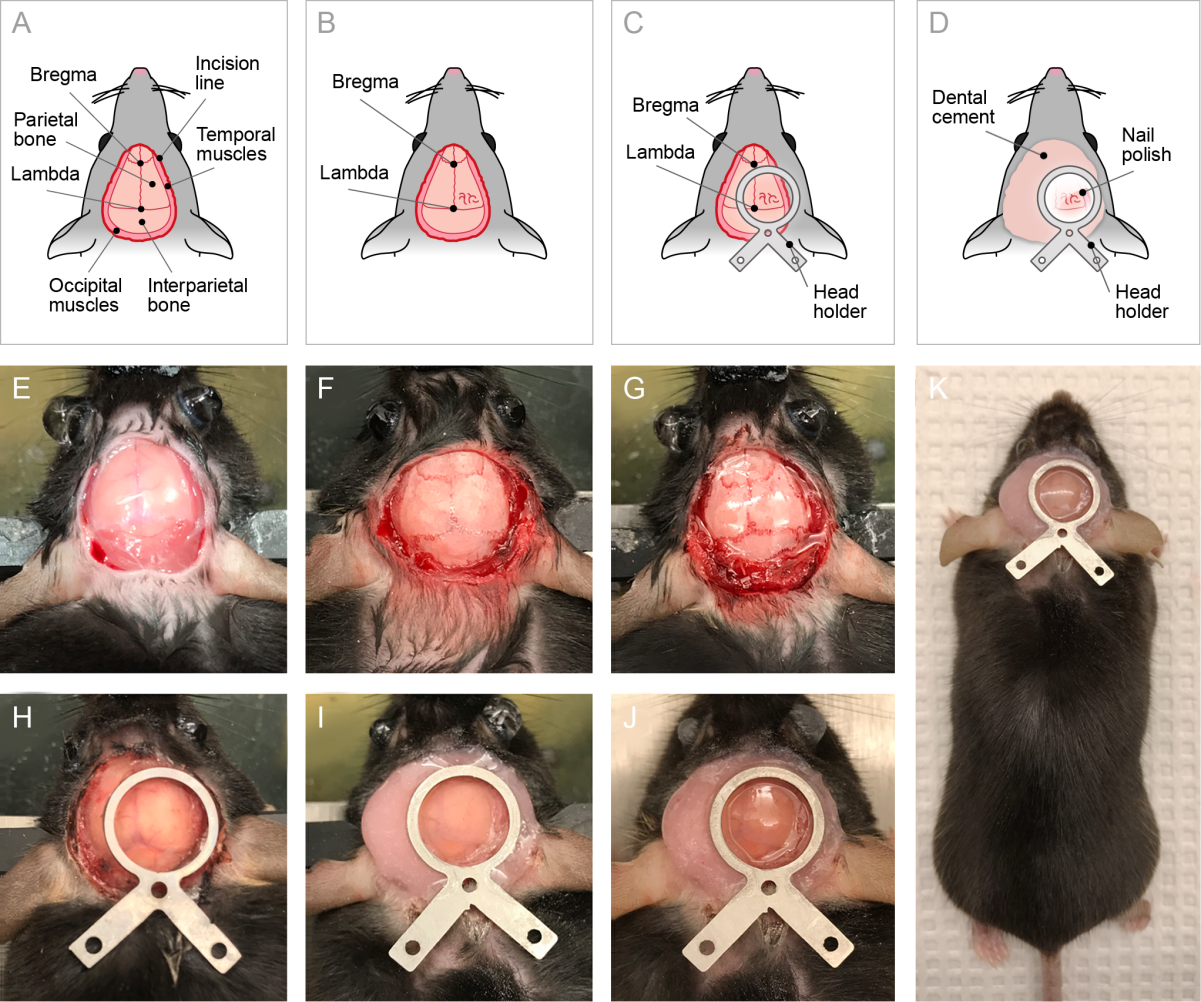
Procedure for TS surgery in mice. (A-D). Schematic view of experimental preparations: (A) Dorsal view on the mouse head with important landmarks and indication of the incision area; (B) Skull after clearing with cyanoacrylate glue obtained increased transparency; (C) Head holder implantation scheme; (D) Nail polish and dental cement application scheme. (E-J). Photographs of the TS surgery main steps (see the protocol for detailed description): (E) Skull after scalp incision (step 8 in the protocol); (F) Cleaned bone surface (steps 9–10); (G) Skull covered with cyanoacrylate glue and acryl (steps 14–19); (H) Head holder mounting (step 24); (I) Head holder fixation with dental cement (step 26); (J) Nail polish added to the window (step 27). (K) Overview of a mouse after the TS surgery.

2 Anesthetize the animal with i.p. injection of 0.1 ml of the mixture per 10 g of mouse weight.
3 Shave the animal's head from the neck to the eye line.
4 Ensure surgical state of anesthesia by checking toe and tail reflexes.
5 Place the animal in a stereotaxic frame on a heating pad to maintain body temperature at 37°C.
6 Protect the eyes with eye gel. Disinfect skin on head with ethanol and Betadine.
7 Dissect the skin around the ROI with spring scissors. Restrict the incision area within 2 mm above the bregma and caudal edge of the interparietal bone. Parietal bones should be fully exposed and temporal and occipital muscles should be visible (Fig 1A).
8 Remove the scalp. Apply a mixture of lidocaine and adrenaline on the incised area for local analgesia and bleeding control. If bleeding continues, use a focused air stream to stop it.
9 Remove periosteum from the skull surface with cotton swabs and an eye scalpel.
10 Push aside the temporal muscles (cut the muscles if necessary) with the eye scalpel. Expose the caudal portion of the temporal ridge on the side of the ROI to create sufficient space to mount the head holder.
11 Gently scrape the surface with the eye scalpel to remove the remaining connective tissue down to white and smooth skull.
12 Clean the skull surface with a cotton swab and dry with air. The surface should be completely dry and clean.

**TIPS**: Do not scratch the bone, as it can damage the skull and cause bleeding.

13 Remove fat from the skull with a cotton swab soaked in acetone. Use air current simultaneously to ensure rapid acetone drying and to prevent acetone penetration into the bone.
14 Apply a thin layer of cyanoacrylate glue to the skull surface using a metal stick (Fig 1B).
15 Wait until the glue dries thoroughly (approximately 10 minutes).
16 Mix acryl powder with methacrylate liquid and stir until nail-polish consistency.

**TIPS:** Add powder to the liquid little by little, to facilitate the dilution and the optimal ratio. Excessive liquid will result in a mixture that will dry too slowly and be too viscous, making it difficult to spread on the skull. Prepare the mixture in advance (10–15 min before application) to allow the acryl to dissolve completely. When properly diluted, the acrylic layer is transparent, homogeneous, and lacking acrylic powder inclusions.

17 Apply the first layer of acryl on the skull surface with a brush.

**TIPS:** Clean the brush with a paper tissue and place in acetone.

18 Wait until the first layer dries (approximately 30 minutes).
19 Apply the second layer and leave it overnight for complete drying.
20 Inject animal with 5–10 mg/kg Carprofen s.c. for postoperative analgesia.
21 Return the animal to its home cage, place the cage on a heating mat for 3–4 hours or overnight to prevent hypothermia.

**TIPS:** Leave only a minimal amount of bedding on the cage floor for this night. Remove all additional bedding and nesting material (especially cotton or other dust-producing materials) to prevent contamination until the acryl layer is thoroughly dried.

#### Steps: day 2

22 Polish the acrylic layer with a hand drill with a polishing bit to achieve a smooth and even surface. Polish only in the ROI. Use air current continuously in the area of polishing to cool the surface.

**TIPS:** Polish very gently, without force, to avoid complete removal of the acryl layer and bone damage. The duration of polishing depends on the acryl layer quality. If the acryl layer is thin, transparent, and does not contain any inclusions, 15–30 seconds of polishing is normally sufficient. In some cases, up to several minutes may be required.

23 Blow away dust and debris with air current. Continue with swabbing if needed.

**TIPS:** Try to achieve a very clean surface to improve adhesion of the head holder.

24 Mount a head holder using a thin layer of the gel glue and wait 5–10 minutes. Try to keep the ROI in the center of the holder (Fig 1C and 1H).

**TIPS**: Do not use too much glue, as excess glue may spread into the ROI.

25 Mix dental cement with cyanoacrylate glue.
26 Cover the skull surface with a thick layer of the dental cement. Place the cement only around the head holder (Fig 1D and 1I).

**TIPS:** Do not overdilute the cement. Watch out for the animal’s eyes, since overdiluted dental cement can easily spread into the eyes.

27 Put transparent nail polish inside the window of the metal holder, above the ROI. This will reduce light scattering and protect the polished surface (Fig 1D and 1J).
28 Keep the animals on the heating mat for at least 3–4 hours after surgery to prevent hypothermia.

## Results

**Fig 2:**
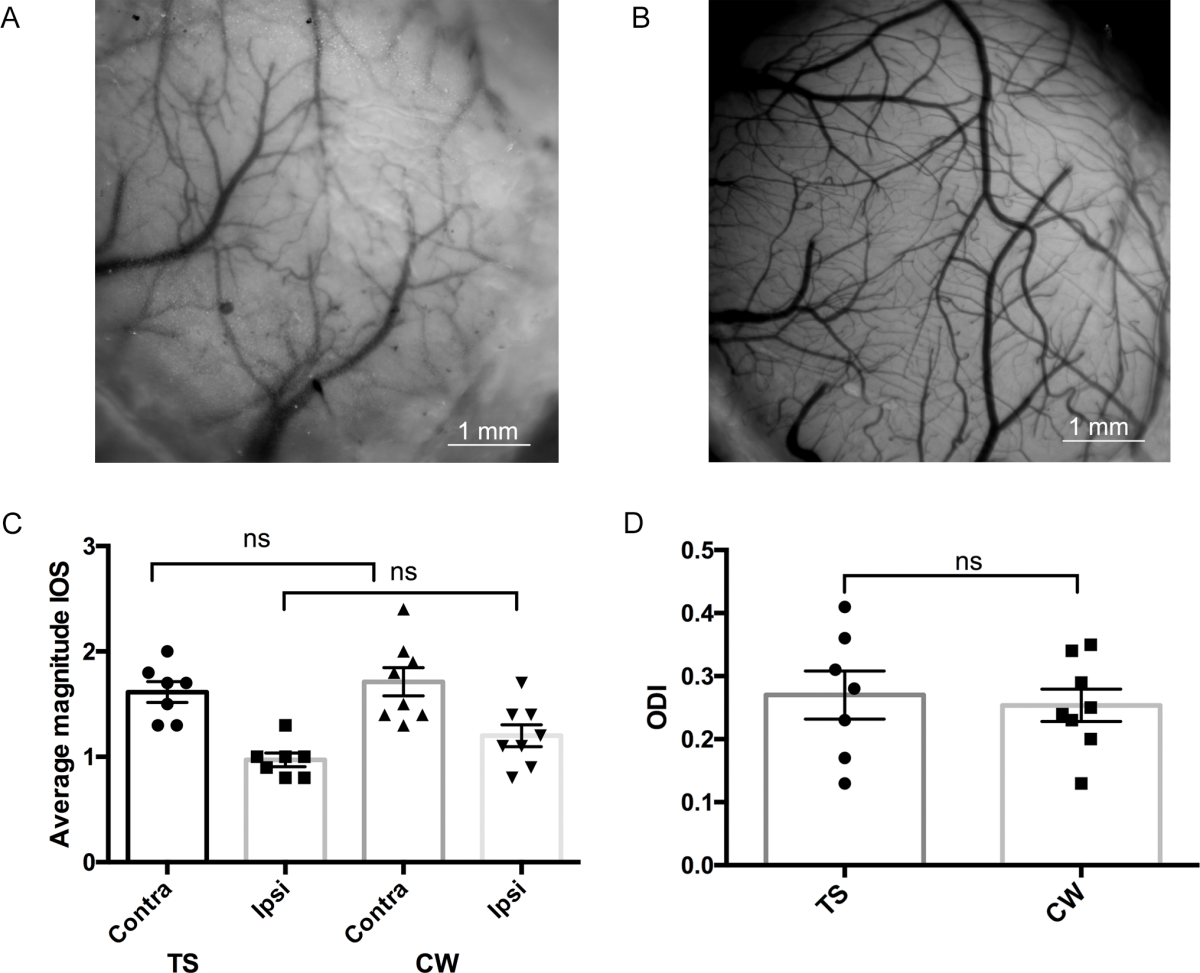
Comparison between properties of the TS and CW in optical imaging experiments. (A) Mouse skull with TS preparation providing a visible vascular pattern of the cortical surface. (B) Mouse skull with conventional CW. (C) The average magnitude of the intrinsic signal measured through TS and CW showed no significant differences for the ipsilateral or contralateral eye (two-way ANOVA, F _1,26_ = 2,365 for the factor “Method”). (D) The ODI measuring in TS and CW groups demonstrated no differences (TS: 0.27±0.03, n = 7; CW: 0.25±0.03, n = 8; t test, P>0.05).

The relative transparency of mouse cranial bones (demonstrated in Fig 2A) allows imaging through the intact skull [22]. As implantation of a cranial window (CW) has certain disadvantages (invasiveness, inflexibility) and requires substantial surgical skills, we took advantage of this skull transparency and developed a skull preparation technique we call here the “transparent skull” (TS) (for detailed protocol see Materials and Methods). This method is characterized by i) relative non-invasiveness, ii) visibility of large cortical areas, iii) sufficient optical transparency, iv) protection of bones from regrowth and inflammation, and v) accessibility for injections. Therefore, the TS meets the desirable criteria for a chronic imaging preparation.

To confirm the applicability of the TS method in practice, we generated two groups of mice: one with a conventional CW and another with TS. We then tested the signal obtained through TS (Fig 2A) and CW (Fig 2B) with the intrinsic signal optical imaging (IOS) method one-week post surgery. As an indirect measure of signal quality, we used a magnitude component of the intrinsic signal. This parameter is based on fractional changes in the reflectance of the red light from the cortical surface, underlying neuronal activity, and represents the activation in the primary visual cortex (V1) evoked by the visual stimulation.

Our optical imaging data showed that the signal magnitude for the ipsilateral (TS: 0.97±0.06, n = 7; CW: 1.19±0.09, n = 8; ANOVA, P>0.05) and the contralateral eye (TS: 1.62±0.1, n = 7; CW: 1.74±0.12, n = 8; ANOVA, P>0.05) did not differ significantly between the two skull preparation techniques (Fig 2C). Statistical analysis details are listed in S1 Table.

Next, we calculated the ocular dominance indexes (ODI) for the animals with TS and a CW. The ODIs assessed through these two skull-preparative procedures showed no significant difference (average ODI, TS: 0.27±0.03, n = 7; CW ODI 0.25±0.03, n = 8; t test, P>0.05) (Fig 2D).

**Fig 3:**
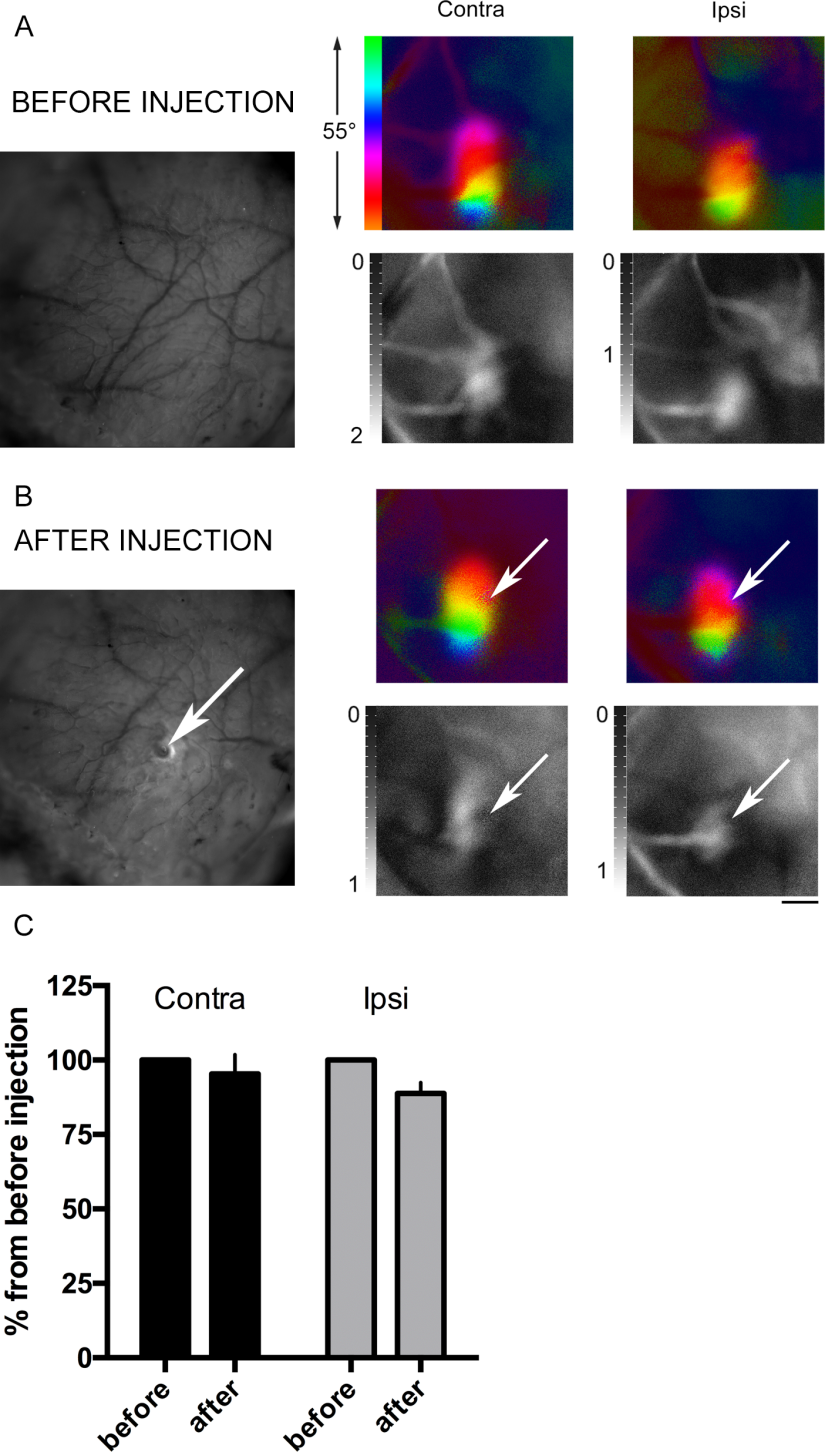
Transparent skull preparation allows injections through the skull. (A) Images of the intact TS. Left: surface vasculature photograph. Right: representative optical imaging data (color images–phase map of retinotopy; b/w images–magnitude map of intrinsic signal). (B) Corresponding images after saline injection through the TS. The injection site is indicated with arrows. Scale bar, 1 mm. (C) IOS data for post-injection signal normalized to the signal before skull drilling and injection (two-way ANOVA (F _1, 10_ = 4.591; P>0,05 for the factor “Injection”).

To test the possibility of injections into the ROI through the TS, we drilled a small hole that allows injection of viruses, drugs, dyes, and other substances. We injected 0.5 μl of saline into the brain parenchyma and then measured the IOS signal. The IOS data demonstrated that saline injection through the TS into the brain parenchyma caused no disruption of the intrinsic signal quality after the procedure (Fig 3A and 3B). Thus, the average magnitude was 96% from the pre-injection signal for the contralateral eye and 86% for the ipsilateral. Two-way ANOVA analysis demonstrated no significant changes for the factor “Injection” (F _1, 10_ = 4.591; *P*>0,05) (Fig 3C).

**Fig 4:**
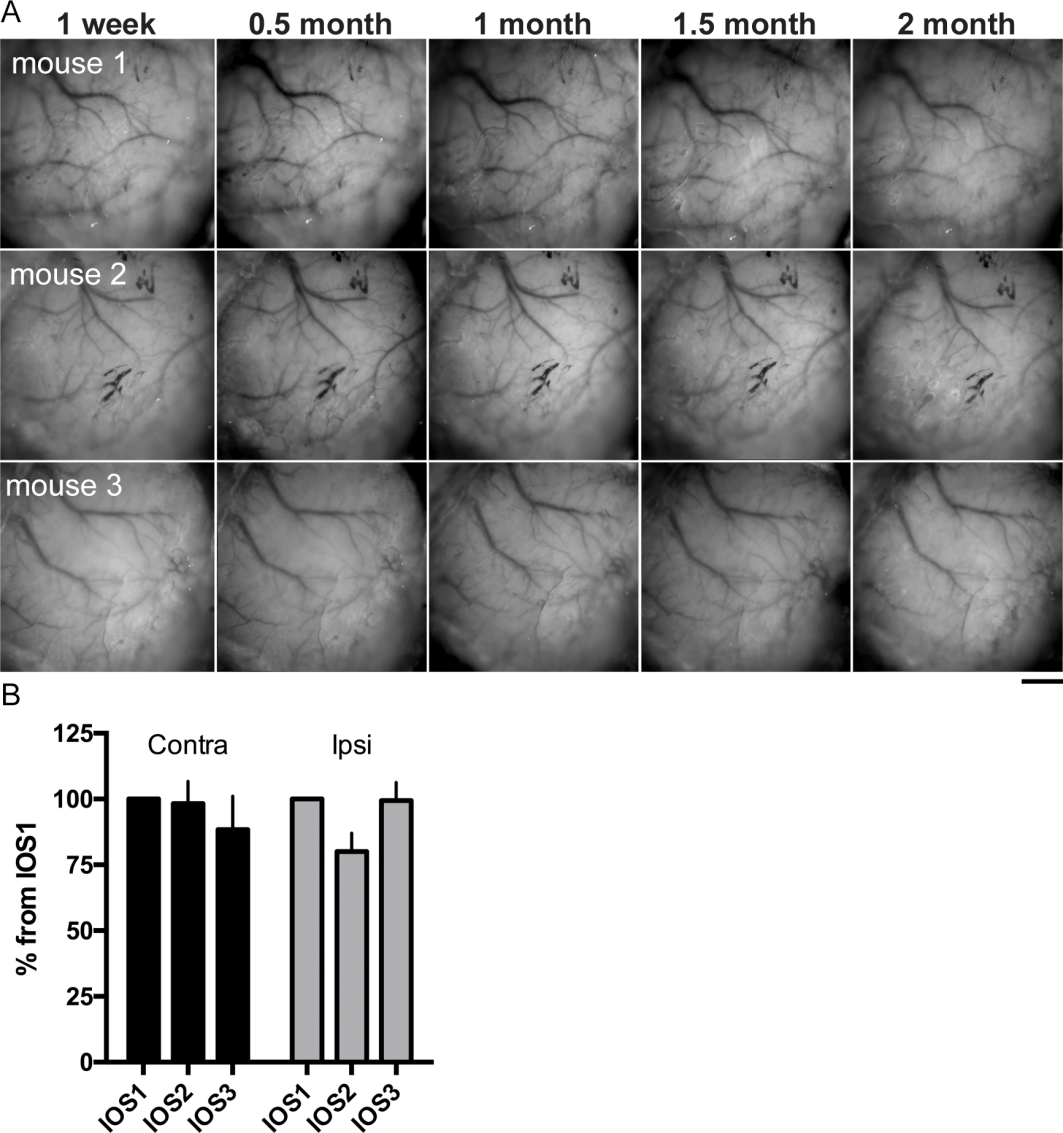
Longitudinal observations of skull transparency and intrinsic signal quality in mice with TS. Mice with TS demonstrated neither significant decline in vascular pattern visibility nor in signal strength (recorded via intrinsic signal optical imaging) within 2–3 months after surgery. (A) Photographs of the skull surface at different time points: 1 week, 0.5 month, 1 month, 1.5 months, and 2 months after surgery. (B) Changes in average magnitude of the intrinsic signals measured through the contralateral and ipsilateral eye–IOS 1: 0–2 weeks; IOS 2: 1.5 months; IOS 3: 2–3 months after TS surgery, normalized to the first imaging–were not significantly different (two-way ANOVA, F _2, 16_ = 0.8925; P>0,05 for the factor “Time”). Scale bar, 1 mm.

To verify the TS applicability to longitudinal studies, we intermittently monitored the skull transparency for two months post-surgery and measured the intrinsic signal response magnitude at different time points. No visible decline in transparency was found during this period (Fig 4A). The signal magnitude of the intrinsic signal experienced a minor decline soon after the surgery, but then remained stable for at least two months with only a slight trend towards diminishing over time. Thus, the average magnitude 2–3 months post-surgery dropped to 88% from the first imaging for the contralateral eye and to 99% for the ipsilateral (Fig 4B). Two-way ANOVA analysis demonstrated no significant changes for the factor “Time” (F 2_, 16_ = 0.8925; *P*>0,05).

**Fig 5:**
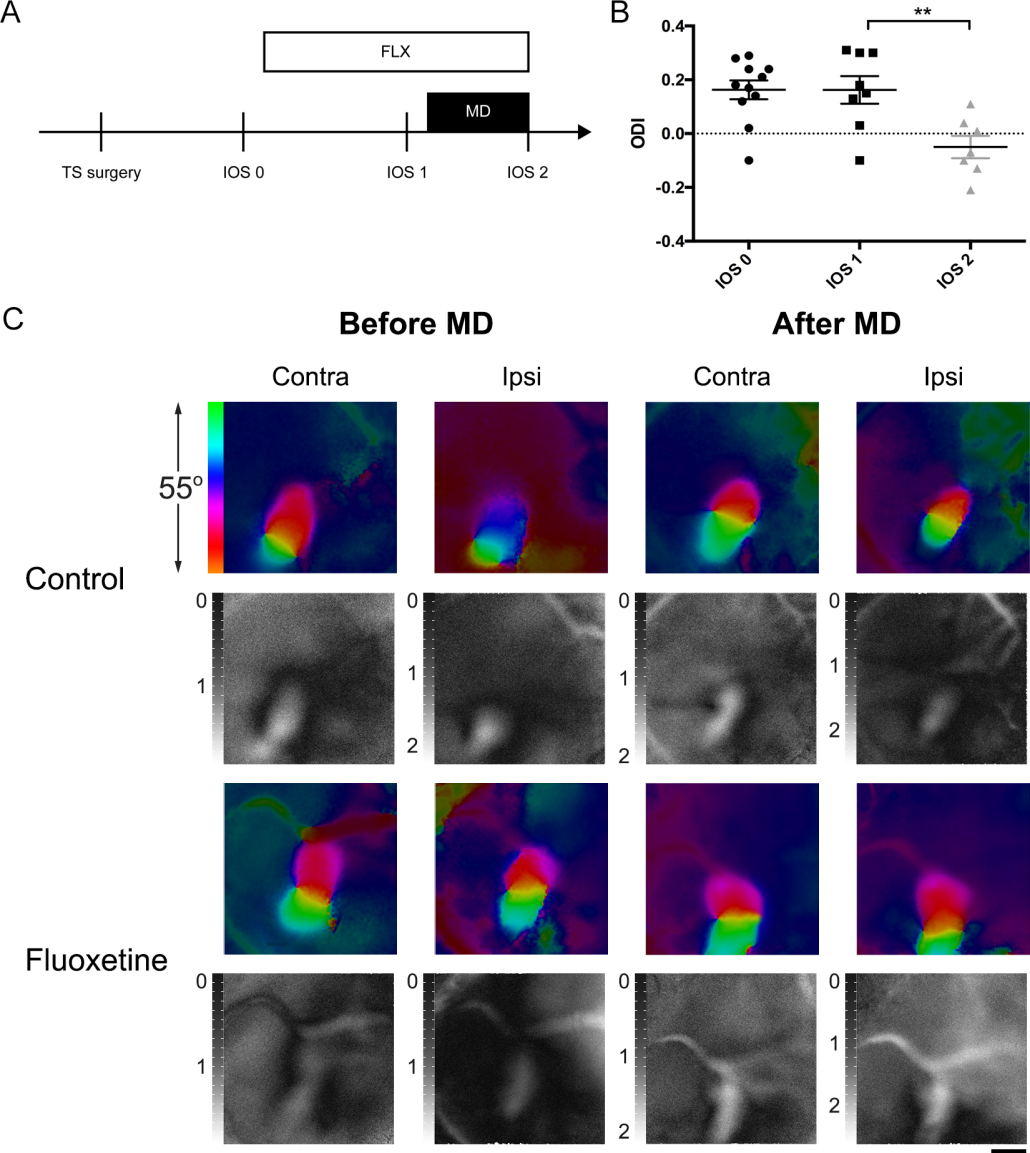
Ocular dominance plasticity restored in adult mice after chronic fluoxetine treatment. (A) Experimental timeline: the first imaging session (IOS 0) was performed before fluoxetine treatment, the next (IOS 1) before monocular deprivation, and the last (IOS 2) after 1 week of monocular deprivation (MD). (B) Chronic 4-week fluoxetine treatment combined with 1-week MD caused a dramatic decline in ODI (from 0.16±0.04, n = 8 to -0.05±0.04, n = 7; ANOVA, P<0.01), whereas fluoxetine treatment without MD failed to produce an effect on OD (IOS 0: 0.16 ± 0.04; IOS 1: 0.16 ± 0.04; one-way ANOVA, P>0.05). (C) Representative IOS data in fluoxetine-treated and water-treated groups before and after monocular deprivation (color images–phase map of retinotopy; b/w images–magnitude map of intrinsic signal). Activity patch of the eye contralateral is normally larger; the ipsilateral patch is smaller. After fluoxetine treatment combined with MD, the patches were similar in size due to weakening of the response to the closed contralateral eye. In controls after monocular deprivation, the patch size remained unchanged. Scale bar, 1 mm.

To further validate the method, we used the TS preparation in experiments with antidepressant-induced plasticity. To induce plasticity in adult visual cortices (beyond PD110) [16], we treated mice for 4 weeks with fluoxetine dissolved in drinking water. During the last week of treatment, the mice underwent monocular deprivation of the eye contralateral to the imaged hemisphere, as previously described for rats [17]. We performed the following three imaging sessions: IOS 0—before the start of fluoxetine treatment, IOS 1 –after the start of the treatment but before monocular deprivation, and IOS 2—after 1 week of monocular deprivation (Fig 5A).

No difference in ODIs before and after fluoxetine treatment was observed, indicating no effect of fluoxetine per se on binocularity and plasticity (ODIs in the fluoxetine-treated group, IOS 0: 0.16 ± 0.04; IOS 1: 0.16 ± 0.04; ANOVA, *P*>0.05) (Fig 5B and 5C).

However, fluoxetine treatment for 4 weeks combined with 7 days of monocular deprivation during the fourth week in adult mice showed a robust shift in OD towards the open eye (ODI IOS 1: 0.16±0.04, n = 8 to IOS 2: -0.05±0.04, n = 7; ANOVA, *P*<0.01) (Fig 5B and 5C). As reported previously, in our water-treated control group monocular deprivation failed to cause a shift in ocular dominance (S2 Fig).

## Discussion

Procedures that allow repeated imaging of brain signals in the same animal over prolonged time periods provide new opportunities to investigate the effects of environmental, genetic, or pharmacological manipulations on brain plasticity. These investigations are not possible with acute recording methods [3,19,23]. Here, we describe the TS technique, a surgical method for skull preparation for chronic optical imaging of intrinsic cortical signals.

In comparing the efficacy of the TS method to CW implantation and further testing it by exploring the fluoxetine-induced critical-period-like plasticity of the mouse visual cortex, we demonstrated that results of the intrinsic optical imaging experiments performed in TS mice are comparable to those obtained with an implanted CW. The signal quality obtained with both methods was also indistinguishable between the two methods.

The TS technique is an inexpensive and simple method for long-lasting, repeated imaging of the visual cortex within a single animal. In addition, this method is relatively non-invasive, avoids damage to the dura mater and minimizes immune system activation, thereby lowering mortality rates.

The surgical procedures required for this method are relatively rapid and easy to learn, which allows simultaneous preparation of a large number of animals. Furthermore, the first imaging session can be performed immediately after surgery, whereas a clearing period of several days is typically required before imaging after CW implantation [7]. Finally, a small hole for the injection of dyes or viral vectors can be drilled onto the TS without compromising visibility.

When compared to earlier alternative methods to CW implantation, our method is maintenance-free and requires no additional preparation immediately before an imaging session, such as polishing or thinning [7,8,11,12].

Possible limitations of this method may include lower optical resolution and possibly diminishing transparency during substantially longer experiments (such as those longer than two months). However, within two months, we observed only a slight decline in intrinsic signal quality transparency.

We used the TS preparation to demonstrate that administration of the antidepressant fluoxetine is able to restore a critical-period-like OD plasticity in adult mice, similar to rats [17]. Our data confirm that chronic fluoxetine treatment reopens a window of critical period-like plasticity in the mouse visual cortex at the time point when natural plasticity is completely absent, and even long-term monocular deprivation fails to promote an OD shift [16]. The efficacy of chronic fluoxetine in promoting an OD shift towards the open eye in mice is similar to that observed after environmental enrichment in rats [20] and mice [19].

We confirmed the effectiveness of the TS method for intrinsic signal optical recordings. Considering the many other approaches to brain imaging, the applicability of the TS for imaging methods such as two-photon imaging warrants further study.

## Supporting information

S1 Fig Monitoring of water consumption. Water drinking in fluoxetine-treated animals was not reduced in comparison to the control group (t test; *P*>0.05).
(TIF)

S2 Fig Ocular dominance measurement in control group. ODI indexes were compared in the same animals before and after monocular deprivation. Water-treated adult mice showed no shift in ocular dominance after one week of monocular deprivation (paired t test, *P*>0.05).
(TIF)

S1 Table Statistical analysis details. (XLSX)
