# Supplementary figures and images for: Chronic imaging through “transparent skull” in mice
Source: PLoS One. 2017 Aug 16;12(8):e0181788. doi: 10.1371/journal.pone.0181788 (PMC5559068; doi:10.1371/journal.pone.0181788)

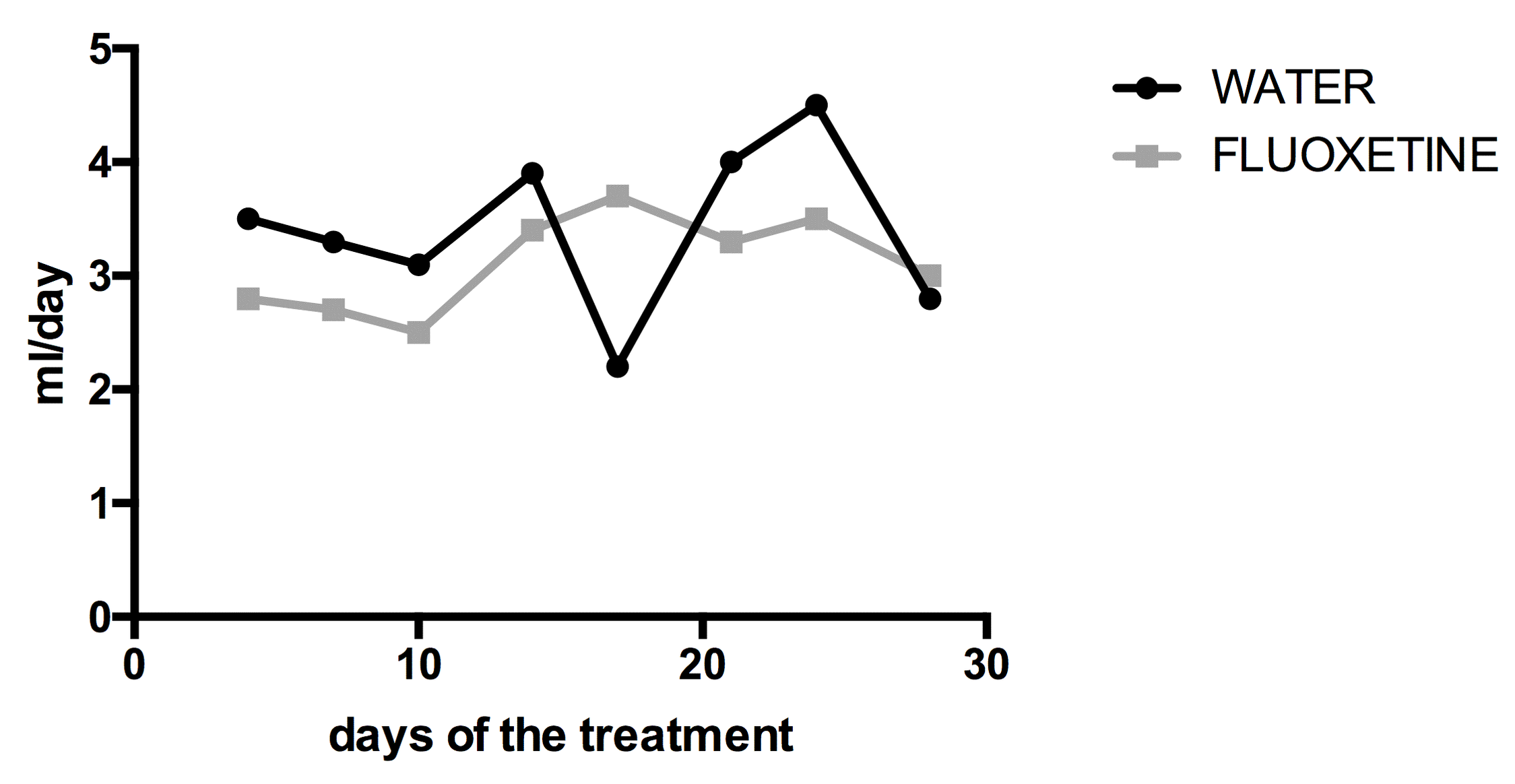

Supplement: S1 Fig — Water drinking in fluoxetine-treated animals was not reduced in comparison to the control group (t test; P>0.05). (TIF) [file pone.0181788.s001.tif]

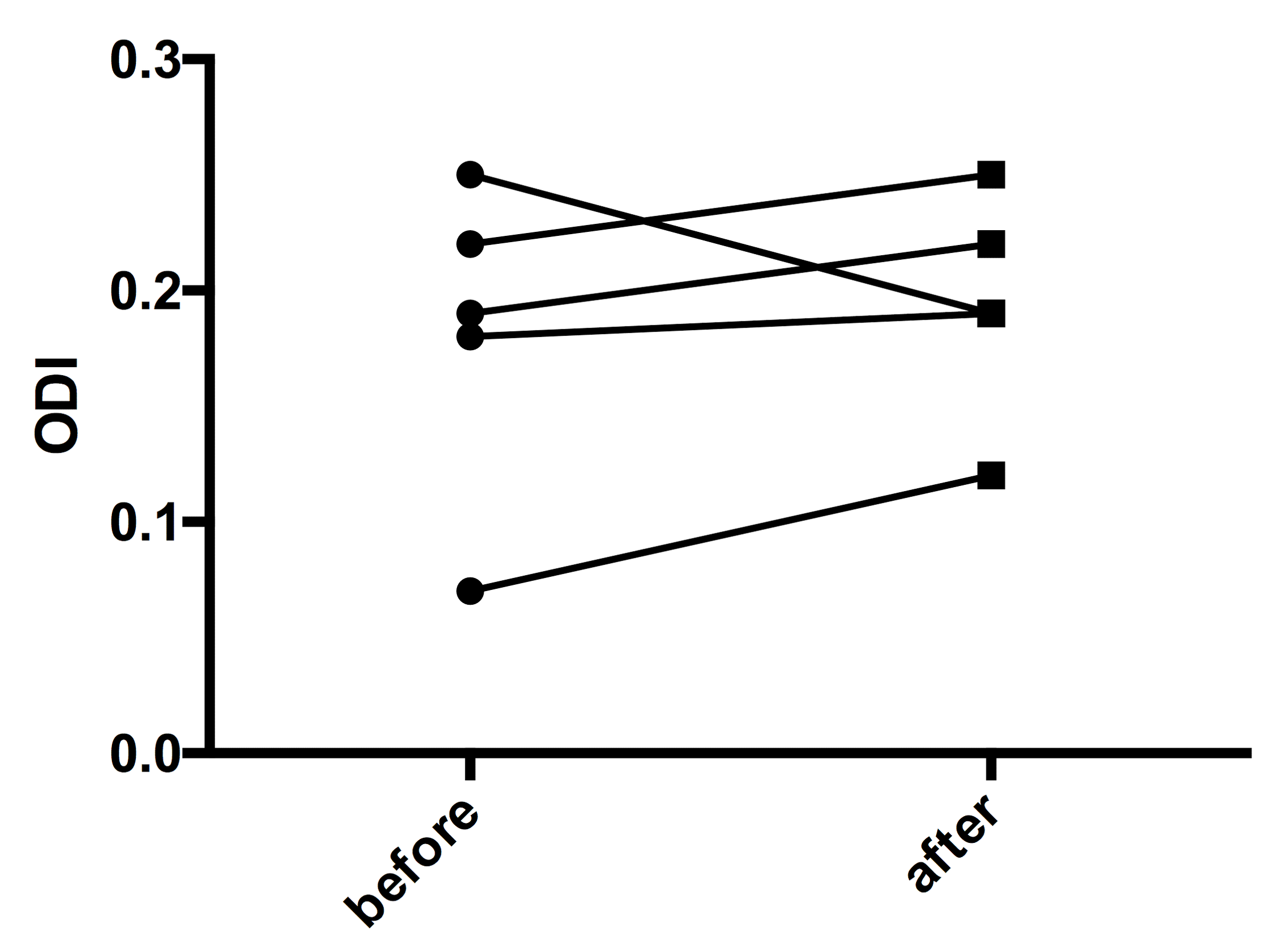

Supplement: S2 Fig — ODI indexes were compared in the same animals before and after monocular deprivation. Water-treated adult mice showed no shift in ocular dominance after one week of monocular deprivation (paired t test, P>0.05). (TIF) [file pone.0181788.s002.tif]
